# Salivary gland function in nasopharyngeal carcinoma before and late after intensity-modulated radiotherapy evaluated by dynamic diffusion-weighted MR imaging with gustatory stimulation

**DOI:** 10.1186/s12903-019-0951-x

**Published:** 2019-12-21

**Authors:** Dai Shi, Jian-Jun Qian, Guo-Hua Fan, Jun-Kang Shen, Ye Tian, Liang Xu

**Affiliations:** 1 Department of Radiology, The Second Affiliated Hospital of Soochow University, Suzhou, 215004 People’s Republic of China; 2 Department of Radiotherapy and Oncolog, The Second Affiliated Hospital of Soochow University, Suzhou, 215004 People’s Republic of China

**Keywords:** Salivary glands function, Diffusion-weighted MR imaging, Nasopharyngeal carcinoma, Radiotherapy, Xerostomia

## Abstract

**Background:**

Xerostomia caused by radiation-induced salivary glands injury has a considerable impact on patients’ quality of life. Nowadays, the existed different methods of evaluating xerostomia in clinical practice there are still some disadvantages and limitations. This study used diffusion-weighted magnetic resonance imaging (DW-MRI) with gustatory stimulation to assess salivary glands function after intensity-modulated radiotherapy (IMRT) in patients with nasopharyngeal carcinoma (NPC).

**Methods:**

DW-MRI was performed in 30 NPC patients and swab method was used to calculate rest and stimulated salivary flow rates (SFR). DW sequence at rest and then repeated ten times during stimulation were obtained. Apparent diffusion coefficients (ADCs) maps of three glands were calculated. Patients before and after RT were recorded as xerostomia and non-xerostomia groups separately. Rest and stimulated ADCs, ADCs increase rates (IRs), time to maximum ADCs (Tmax), ADCs change rates (CRs), rest and stimulated SFR, SFR increase rates (IRs) and SFR change rates (CRs) before and after RT were assessed.

**Results:**

The rest and stimulated ADCs of three glands after RT were higher than those before RT (*p* < 0.001). The rest and stimulated SFR of all salivary glands after RT were lower than those before RT (*p* < 0.001). A correlation existed between rest ADCs of submandibular glands and rest SFR of submandibular mixed with sublingual glands and full three glands before RT (*p* = 0.019, *p* = 0.009), stimulated ADCs and stimulated SFR in parotid glands before RT (*p* = 0.047). The rest ADCs of parotid glands after RT correlated to XQ scores (*p* = 0.037).

**Conclusions:**

The salivary glands’ ADCs increased after RT both in rest and stimulated state due to the radiation injury and the ADCs correlated with SFR and XQ scores of evaluating the xerostomia in clinical practice.

## Background

Xerostomia is an important complication results from radiation-induced salivary glands injury, which has a considerable impact on patients’ quality of life [1–4]. Nowadays, there are still some disadvantages and limitations of the existed different methods of evaluating xerostomia in clinical practice [5–9]. In addition, different methods of evaluation are inconsistent, resulting in a worse comparability and reproducibility. Therefore, an effective method, especially noninvasively, to evaluate salivary glands function after radiotherapy (RT) is demanded.

Diffusion-weighted magnetic resonance imaging (DW-MRI), a noninvasive imaging technique, allows the depiction of molecular diffusion caused by Brownian motion in biological tissues. The water molecule diffusion leads to signal attenuation, which can be quantified as the apparent diffusion coefficients (ADCs) [10–12]. The rest and stimulated ADCs represent the basic and reserve function of salivary glands separately, so the ADCs before and after stimulation can be used to fully evaluate the functional status of salivary glands. Many studies [10, 13–20] have suggested that DW-MRI based on the glands’ morphological and functional changes [14, 18, 20–22] has great potential in investigating the mechanisms of the salivary glands injury induced by radiation. However, the salivary glands’ pattern of reaction to gustatory stimulation in DW-MRI and changes in ADCs as a result of RT have no consistent results in previous studies [10, 13–15, 23–25]. And for the evaluation of xerostomia induced by RT, DW-MRI has been used to assess the parotid glands [13, 15, 26] and submandibular glands [20, 27, 28] based on rest and stimulated ADCs, but the sublingual glands have not been assessed. Another concern is that the relationship between the ADCs and clinical methods used for evaluation of xerostomia, such as salivary flow rates (SFR) and xerostomia questionnaire (XQ) scores were also remains unclear and have not been proven consistent previously [15]. Moreover, the thresholds of ADCs and SFR suggesting xerostomia are still lacking.

In our prospective study of 30 nasopharyngeal carcinoma (NPC) patients treated with intensity-modulated radiotherapy (IMRT), we evaluated the feasibility of dynamic DW-MRI used transient gustatory stimulation with lemon juice in evaluation of salivary glands function before and late after IMRT by compared and correlated the changes in ADCs with clinical methods, being the SFR and the XQ scores, for evaluation of xerostomia. We hypothesized that the ADCs correlated with SFR and XQ scores, which suggested the ADCs can reflect the salivary glands injury induced by RT accurately.

## Methods

### Participants

The study protocol was approved by the Institutional Ethical Committee of our hospital and informed consent was obtained from all patients. Patients who were first diagnosed and undergone IMRT in the radiotherapy department between September 2011 and December 2016 were recruited. The inclusion criteria were, (1) the patients’ condition meets the MRI examination requirements, (2) KPS score > 70 before RT, (3) patients were the first time accepted IMRT and without distant metastasis, (4) patients diagnosed as NPC pathologically. The exclusion criteria were, (1) patients with salivary glands disease, (2) patients received chemoradiotherapy before, (3) patients with systemic diseases such as Sjogren syndrome, malignant lymphoma, diabetes, (4) patients with Alzheimer’s disease and stroke, (5) patients with chronic drug history, (6) patients with history of smoking and alcoholism.

According to the inclusion and exclusion criteria, 30 patients (21 men and 9 women, age range, 38–71 years, median age, 57 years) were recruited. The follow-up time was 12–30 months after RT, the mean length follow-up time was 16.6 months. All patients completed a XQ scores established by the University of Michigan in the United States (Table 1) [29]. All patients were completed the expected RT and received systemic chemotherapy based on platinum or platinum drugs during RT and underwent DW-MRI before and after RT (Additional file 1).

**Table 1:** The xerostomia questionnaire (XQ) scores

1. Rate your difficulty in talking due to dryness	
2. Rate your difficulty in chewing due to dryness	
3. Rate your difficulty in swallowing solid food due to dryness	
4. Rate the frequency of your sleeping problems due to dryness	
5. Rate your mouth or throat dryness when eating food	
6. Rate your mouth or throat dryness while not eating	
7. Rate the frequency of sipping liquids to aid swallowing food	
8. Rate the frequency of sipping liquids for oral comfort when not eating	

In this study, all patients had no xerostomia before RT and all of them occurred to xerostomia after RT, so we recorded the patients as non-xerostomia group before RT and xerostomia group after RT.

### MR imaging

All MR examinations were performed on a 1.5-T MR system (Intera Achieva 1.5 T Pulsar, Philips Medical Systems, Netherlands) using a 16 channel head-neck coil. The three pairs of salivary glands of all patients undergone DWI before and after RT. All patients were asked not to eat or drink for at least one hour before MRI examination. The images extended from the skull base to the thoracic inlet.

Transverse DWI was obtained using a single-shot echo-planar imaging sequence with the b-value of 0 and 1000 s/mm^2^. DWI (TR = 4000 ms, TE = 55 ms, TI = 170 ms, matrix size = 256 × 512) were obtained with a section thickness of 6 mm and intersection gap of 1 mm, and a 180 field of view. The slice number and data acquisition time of DWI from the parotid to sublingual glands were 26 slices and 60 s. After RT, a simplified DWI protocol with the same parameters was used.

One DWI series was acquired at rest, after which 5 ml of a commercially available unsweetened 100% lemon juice (Lemondor, Italy) was injected into subjects’ mouths, and then instructed subjects to hold the juice in their mouths for exactly 10 s before swallowing. Thirty seconds after injecting the lemon juice, a set of 10 DWI were obtained with a time interval of 30 s between consecutive repeats and one minute 30 s for once sequence.

### MR image data analysis

The original data of DWI was processed by using the Extended MR Work Space software of Philips. An experienced neuroradiologist reviewed all images and defined regions of interests (ROIs). On the native DW images, ROIs were drawn freehand around the parotid glands, submandibular glands and sublingual glands on all sections. Regions containing large vessels such as retromandibular vein and external carotid artery were excluded, the signal intensity (SI) of every salivary gland of every section was calculated, and then took the average SI of each gland as the glands’ SI. ADCs was calculated from the equation: ADCs = [ln (SI1/SI2)]/(b2-b1) where SI1 and SI2 were the signal intensity measured on the corresponding b value image, b1 and b2 were the corresponding b value. The ADCs was measured on maps using regions if ROIs placed over each of the salivary glands, and the final calculation of ADCs was performed using the average value of each ROI. For the purpose, all of the salivary glands were manually delineated on DWI sequence.

ADCs increase rates (IRs) after stimulation was defined as follows: ADCs IRs(%) = (maximum ADCs after stimulation – ADCs before stimulation) / ADCs before stimulation. Tmax (in seconds) was defined as time to maximum ADCs after stimulation. The ADCs, ADCs IRs and Tmax of each gland separately were calculated. Plotting the ADCs-time curve according to the ADCs of each salivary gland at each time point before and after stimulation, the peak value corresponds to the Tmax and the maximum ADCs after stimulation.

The rest ADCs, maximum ADCs after stimulation, ADCs IRs and Tmax were recorded before and after RT. The ADCs change rates (CRs) at rest and after stimulation before and after RT were calculated separately by the equation: ADCs CRs(%) = (ADCs after RT – ADCs before RT)/ ADCs before RT.

### Method of saliva collection and measurement

In sialometry, the swab method [30] was used, all patients were assessed in the morning, at least one hour after a meal, the mouth was cleaned and dried by gauze before assessment, and the patients were upright position. The dental cotton rolls were positioned at the orifices of the ducts of three salivary glands separately to collect saliva from both sides of the parotid (Stenson’s duct), the submandibular (Wharton’s duct) and the sublingual glands. The dental rolls were placed in a fixed order as quickly as possible, starting from the upper region of the mouth. The endings of the Stenson ducts were covered to prevent saliva from leaking into the lower parts of the oral cavity. In this way, saliva was obtained separately from the different oral regions. The measurements of submandibular and sublingual glands may not be separated because their orifices of ducts have a close anatomic relation, so the compound measurements of these glands was referred to as submandibular mixed with sublingual salivary flow rates. During a 5-min period, the cotton rolls absorbed sufficient saliva so that an increase in weight could be measured.

For the stimulated SFR, 5 ml of a commercially available unsweetened 100% lemon juice was injected into subjects’ mouths, and then instructed subjects to hold the juice in their mouths for exactly 10 s before swallowing, after which the measuring method of stimulated SFR like with the rest above. Recording the rest and stimulated SFR and the changes in SFR of the parotid glands, submandibular mixed with sublingual glands, and the full three glands. The SFR increase rates (IRs) after stimulation was defined as follows: SFR IRs(%) = (maximum SFR after stimulation –SFR before stimulation) / SFR before stimulation. The rest and stimulated SFR change rates (CRs) before and after RT were calculated separately by the equation: SFR CRs(%) = (SFR after RT –SFR s before RT)/ SFR before RT.

### Statistical analysis

All statistical analysis was performed using MedCalc 13.1.2.0 (MedCalc Software, Ostend, Belgium). Continuous variables with normal distribution presented as mean ± SD. The paired two-tailed Student’s t test was used to compare the rest and stimulated ADCs, ADCs IRs, Tmax, the rest and stimulated SFR, SFR IRs and the SFR CRs separately. The one-way ANOVA test was used to compare the ADCs and changes in ADCs among the three glands, the rest and stimulated SFR among the parotid gland, submandibular mixed with sublingual and the full three glands before and after RT respectively. The Pearson’s rank correlation coefficient was calculated to correlate the changes in ADCs and changes in SFR and XQ scores respectively. The ROC curve was used to refer the cutoff value of the parameters statistically significant between xerostomia and non-xerostomia groups. A value of *p* < 0.05 was considered significant.

## Results

Thirty NPC patients, 17 cases were low differentiation squamous cell carcinoma, 11 cases were undifferentiated non-keratinized carcinoma, and 2 cases were non-keratinized carcinoma. One, six, 14, nine cases were I, II, III, IV phase respectively.

As a result of stimulation with lemon juice, an initial steady increase to peak during the first DWI scan (30 s) and subsequent decrease in ADCs was seen in all glands before and after RT. The Tmax before RT was statistically significant among the three salivary glands (*p* = 0.008), the Tmax of parotid glands was the earliest. Tmax of sublingual glands after RT was significantly earlier than that before RT (*p* = 0.004). The rest and stimulated ADCs of three glands after RT were all significantly higher than those before RT (*p* = 0.000). For both before and after RT, the rest ADCs was higher in submandibular glands (1.09 ± 0.06, 1.45 ± 0.14) than in parotid glands (0.95 ± 0.08, 1.11 ± 0.16) and sublingual glands (0.94 ± 0.07, 1.25 ± 0.14) (*p* = 0.000) (Table 2).

**Table 2:** The ADCs and ADCs changes of the salivary glands before and after RT

Parameters	PG	SMG	SLG	*F*	*P*
Tmax, pre, (s)	134.00 ± 243.56	310.00 ± 328.00	391.33 ± 368.48	5.145	0.008
Tmax, post, (s)	174.00 ± 240.83	194.00 ± 223.91	158.00 ± 222.69^a^	0.186	0.831
ADCrest,pre(×10^−3^ mm^2^/s)	0.95 ± 0.08	1.09 ± 0.06	0.94 ± 0.07	40.365	0.000
ADCrest,post(×10^−3^ mm^2^/s)	1.11 ± 0.16^b^	1.45 ± 0.14^c^	1.25 ± 0.14^d^	41.768	0.000
ADCstim, pre(×10^−3^ mm^2^/s)	1.04 ± 0.08	1.17 ± 0.09	1.20 ± 0.13	19.266	0.000
ADCstim,post(×10^−3^ mm^2^/s)	1.22 ± 0.18^e^	1.58 ± 0.17^f^	1.61 ± 0.22^g^	38.572	0.000
ADCstim, pre IRs(%)	10.51 ± 6.44	7.35 ± 6.11	28.19 ± 16.61	35.121	0.000
ADCstim,post IRs(%)	10.17 ± 8.33	9.47 ± 9.79	29.15 ± 18.39	22.280	0.000
ADCrest, CRs(%)	17.21 ± 15.32	33.19 ± 13.28	32.94 ± 13.68	12.625	0.000
ADCstim, CRs (%)	17.20 ± 19.28	35.49 ± 14.10	34.22 ± 19.21	9.993	0.000

*ADCrest,pre* the rest ADCs pre-RT, *ADCrest,post* the rest ADCs post-RT, *ADCstim,pre* the stimulated ADCs pre-RT, *ADCstim,post* the stimulated ADCs post-RT, *ADCstim,pre IRs* the maximum stimulated ADCs IRs pre-RT, *ADCstim,post IRs* the maximum stimulated ADCs IRs post-RT, *ADCrest,CRs* the rest ADCs CRs before and after RT, *ADCstim,CRs* the stimulated maximum ADCs CRs before and after RT. *PG* parotid glands, *SMG* submandibular glands; SLG: sublingual glands

Pa = 0.004; Pb = 0.000; Pc = 0.000; Pd = 0.000; Pe = 0.000; Pf = 0.000; Pg = 0.000

Pa~g: the ADCs and ADCs changes of the salivary glands before and after RT had statistical significance

The ADCs IRs in sublingual glands (28.19 ± 16.61, 29.15 ± 18.39) was higher than in parotid glands (10.51 ± 6.44, 10.17 ± 8.33) and submandibular glands (7.35 ± 6.11, 9.47 ± 9.79) before and after RT respectively (*p* = 0.000), and the ADCs IRs in parotid glands (10.51 ± 6.44, 10.17 ± 8.33) was higher than in submandibular glands (7.35 ± 6.11, 9.47 ± 9.89) before and after RT respectively (*p* = 0.000). The ADCs IRs before and after RT had no statistical significance of the three glands respectively (*p*>0.05). Both of the rest and the stimulated ADC CRs had no statistical significance in the three glands respectively(*p*>0.05) (Table 2).

The rest and stimulated SFR of parotid glands, submandibular mixed with sublingual glands and full three glands after RT were lower than those before RT (*p* = 0.000). After stimulation, the SFR IRs of parotid glands and the full three glands after RT (7.30 ± 4.99, 6.07 ± 12.07) were higher than those before RT (2.75 ± 2.34, 3.93 ± 7.68) (*p* = 0.000). The absolute value of the rest SFR CRs in parotid glands and the full three glands were higher (61.60 ± 32.13, 82.46 ± 8.82) than the stimulated SFR CRs (17.82 ± 61.32, 59.84 ± 26.22) (*p* = 0.000). The absolute value of the rest and the stimulated SFR CRs in parotid glands were lower (61.60 ± 32.13, 17.82 ± 61.32) than submandibular mixed with sublingual glands (87.43 ± 14.00, 82.48 ± 16.84) (*p* = 0.000) (Table 3).

**Table 3:** Salivary flow rates and the changes of salivary flow rates of the salivary glands before and after RT

Parameters	PG	SMG / SLG	FG	*F*	*P*
SFRrest, pre(g/min)	0.26 ± 0.17	0.49 ± 0.30	0.76 ± 0.42	17.625	0.000
SFRrest, post(g/min)	0.07 ± 0.03^a^	0.04 ± 0.05^b^	0.12 ± 0.05^c^	25.458	0.000
SFRstim, pre(g/min)	0.72 ± 0.49	1.11 ± 0.51	1.82 ± 0.56	47.114	0.000
SFRstim, post(g/min)	0.54 ± 0.75^d^	0.19 ± 0.32^e^	0.72 ± 0.55^f^	16.053	0.000
SFRstim, IR, pre(%)	2.75 ± 2.34	3.93 ± 7.68	2.55 ± 3.08	0.670	0.514
SFRstim, IR, post(%)	7.30 ± 4.99^*g*^	6.07 ± 12.07	6.63 ± 6.73^*h*^	0.269	0.765
SFRrest, CRs (%)	−61.60 ± 32.13	−87.43 ± 14.00	−82.46 ± 8.82	12.019	0.000
SFRstim, CRs (%)	−17.82 ± 61.32^i^	−82.48 ± 16.84	−59.84 ± 26.22^j^	20.483	0.000

*SFR* salivary flow rates, *SFRrest,pre* the rest salivary flow rates pre-RT, *SFRrest,post* the rest salivary flow rates post-RT, *SFRstim,pre* the stimulated salivary flow rates pre-RT, *SFRstim,post* the stimulated salivary flow rates post-RT, *SFRstim,IR,pre* the stimulated salivary flow rates IRs pre-RT, *SFRstim,IR,post* the stimulated salivary flow rates IRs post-RT, *SFRrest,CRs* the rest salivary flow rates CRs pre and post-RT, *SFRstim,CRs* the stimulated salivary flow rates CRs pre and post-RT. *PG* parotid glands, *SMG / SLG* submandibular glands and sublingual glands, *FG* full of three glands

Pa = 0.000; Pb = 0.000; Pc = 0.000; Pd = 0.023; Pe = 0.000; Pf = 0.000; Pg = 0.002; Ph = 0.007

Pi = 0.001; Pj = 0.001

Pa~j: salivary flow rates and the changes of salivary flow rates of the salivary glands before and after RT had statistical significance

The cutoff value of the rest ADCs in parotid glands, submandibular glands and the sublingual glands between xerostomia and non-xerostomia groups were 1.01, 1.20 and 1.08 (*p* = 0.000). The cutoff value of the stimulated ADCs in parotid glands, submandibular glands and sublingual glands between xerostomia and non-xerostomia groups were 1.10, 1.32 and 1.32 (*p* = 0.000). The cutoff value of the rest and stimulated SFR of full three glands between xerostomia and non-xerostomia groups were 0.19 g/min and 0.79 g/min (*p* = 0.000) (Table 4).

**Table 4:** The ROC curve of rest and stimulated ADCs

Parameters	PG	SMG	SLG
ADCrest, pre-post			
cutoff value(×10^−3^ mm^2^/s)	1.01	1.20	1.08
sensitivity	0.667	0.933	0.900
specificity	0.867	1.000	0.967
Youden index	0.533	0.933	0.867
AUC	0.813	0.989	0.964
ADCstim, pre-post			
cutoff value(×10^−3^ mm^2^/s)	1.10	1.32	1.32
sensitivity	0.800	1.000	0.867
specificity	0.800	0.933	0.867
Youden index	0.600	0.933	0.733
AUC	0.824	0.989	0.949

*ADCrest,pre-post* the rest ADCs before and after RT, *ADCstim,pre-post* the maximum stimulated ADCs before and after RT.

**Fig. 1:**
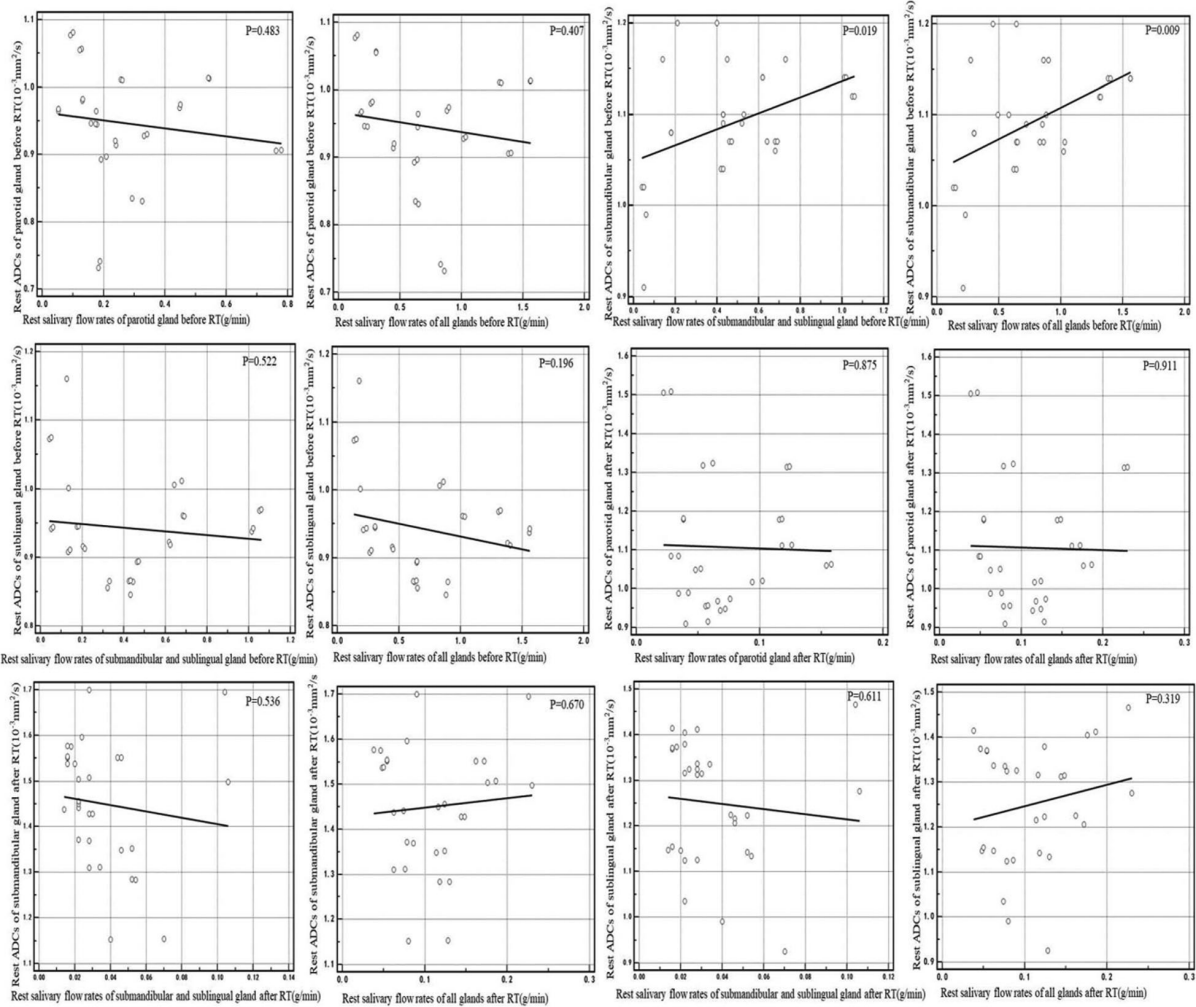
Correlation between the rest ADCs before and after RT and the rest salivary flow rates separately

**Fig. 2:**
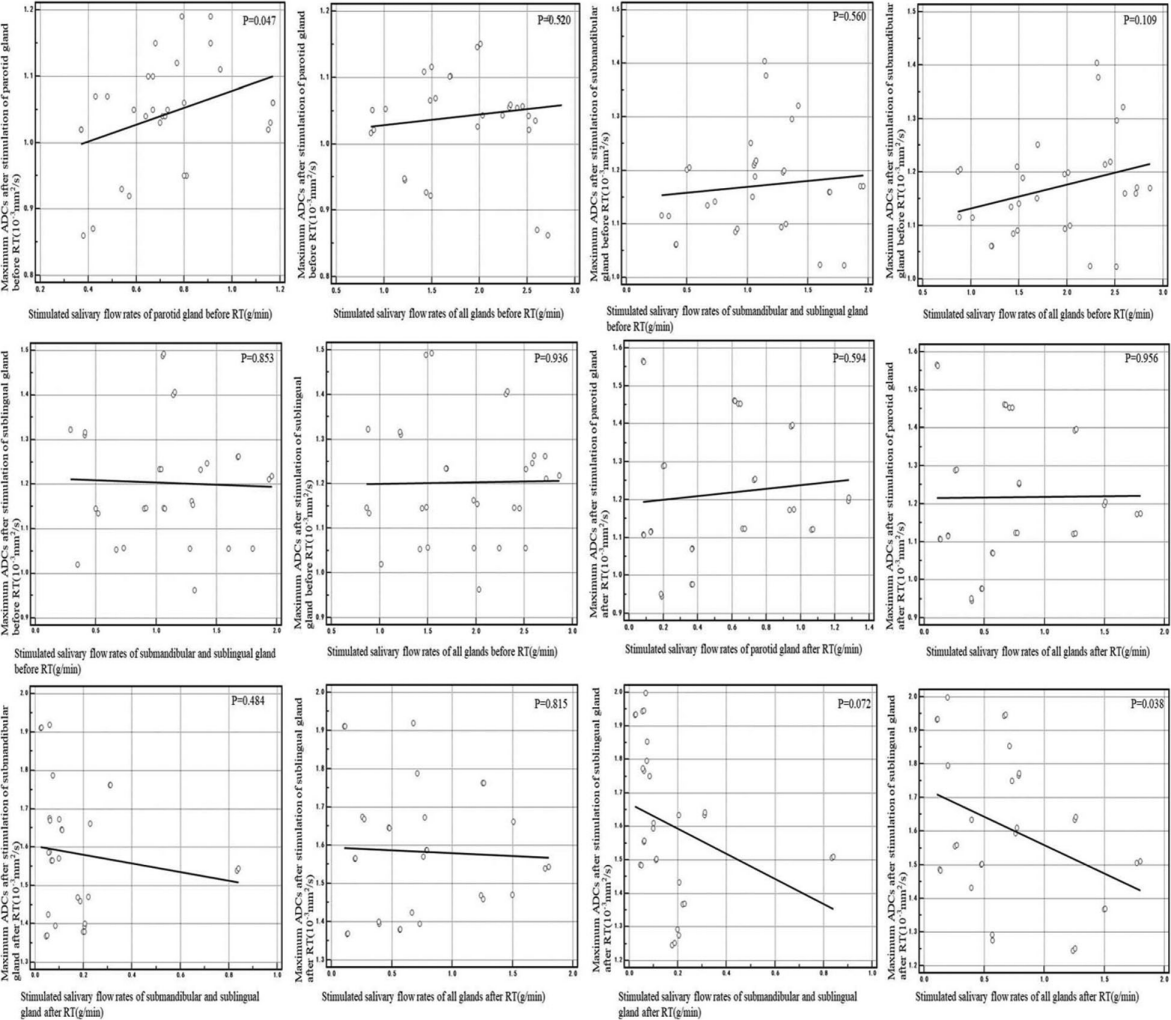
Correlation between the maximum ADCs after stimulation before and after RT and the stimulated salivary flow rates separately

A positive correlation existed between the rest ADCs of submandibular glands and the rest SFR of submandibular mixed with sublingual glands and the full three glands before RT (*r* = 0.425, *p* = 0.019; *r* = 0.470, *p* = 0.009) (Fig. 1). A positive correlation also existed between the stimulated ADCs and the stimulated SFR in parotid glands before RT (r = 0.366, *p* = 0.047)(Fig. 2). A negative correlation also existed between the stimulated ADCs of sublingual glands and the stimulated SFR of full three glands after RT (r = 0.381, *p* = 0.038) (Fig. 2).

**Fig. 3:**
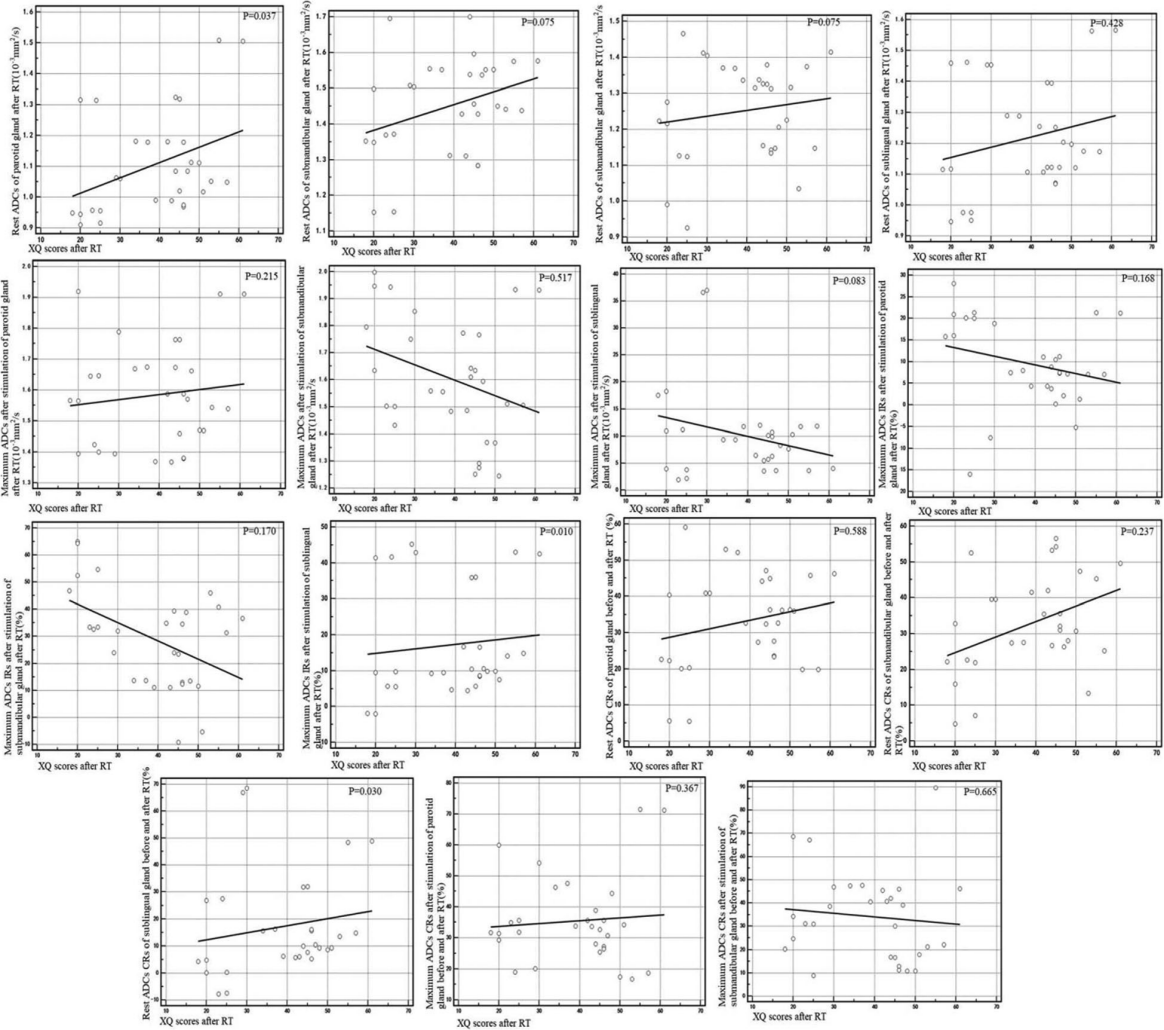
Correlation between the rest ADCs after RT, the maximum stimulated ADCs after RT, the maximum ADCs IRs after RT, the rest ADCs CRs before and after RT, the maximum ADCs CRs after stimulation before and after RT, and the XQ scores after RT separately

For the correlation between ADCs and XQ scores, a positive correlation existed between the rest ADCs of parotid glands after RT and XQ scores (*r* = 0.382, *p* = 0.037), the stimulated ADCs IRs of sublingual after RT and the XQ scores(*r* = 0.462,*p* = 0.010), the rest ADCs CRs of sublingual and the XQ scores(*r* = 0.397, *p* = 0.030)(Fig. 3).

## Discussion

Even though modern radiotherapy techniques, such as the IMRT, allow a dose distribution highly conformal to the target volumes, which spare the salivary glands function after RT is not always possible [10, 31, 32], thus, the reduction of salivary glands function is still a common side effect of RT [33]. Xerostomia is an important complication results from radiation-induced glands injury, which has great impact on quality of life [1–4]. Reduced salivary glands function and declined quality of life related to oral health are typical both in the acute phase (and reversible after the end of therapy) and the chronic phase and sometimes can be lifelong [1, 34]. Messmer et al. found that xerostomia did not change significantly over the period of 5 years based on 121 patients followed for 41–90 months after RT, and both the difficulties with speaking and eating condition worsened [3], the mechanism remains unclear, and the histological changes in the gland tissue during radiation are not accessible in vivo [35, 36]. At the same time, because of the high variability in salivary glands function among patients, it is important and necessary to individually assess and monitor the function of salivary glands using in vivo quantitative tools efficiently [37].

In the major salivary glands, the sublingual gland is the smallest and the measuring and calculation of it is difficult to some extent, which is the reason why the sublingual gland was not included in previous studies. In this prospective study, a DWI protocol was applied to patients with NPC before and late after RT at rest and after stimulation with lemon juice in parotid glands, submandibular glands and sublingual glands respectively. These results were compared with SFR and XQ scores. Our main findings were that an initial steady increase to peak during the first DWI scan and subsequent decrease in ADCs was seen in all glands before and after RT as a result of stimulation with lemon juice, suggesting a persisting capacity of the acinar cells to produce saliva after stimulation. The ADCs IRs before and after RT had no statistical significance in all three glands and the maximum ADCs after stimulation of the three glands were higher than the rest ADCs before and after RT respectively also support this view. To date, only ascorbic acid tablet [10, 14, 24] and lemon juice [23, 25] were used to stimulate the salivary glands. Some researchers [25] have used lemon juice with six tablets of 100 mg as a stimulant and other researchers [13] performed the gustatory stimulation with two 500 mg tablets of ascorbic acid – reported an initial increase and subsequent fluctuation in the ADCs, those results are in accordance with our study. However, Thoeny [38] and Dirix [14] used a 500 mg tablet of ascorbic acid as the stimulant and advised the patients to let the tablet dissolve but not to chew it in their mouths, which resulted in an initial decrease and subsequent increase in the ADCs [24, 39]. According to the previous studies, the pattern of response to gustatory stimulation in salivary glands varied widely. This may contribute to the difference of the species and amount of stimulant. The gustatory stimulation induced by ascorbic acid continuous during MR imaging due to ascorbic acid tablet dissolves completely after a median 23 min (range, 20-27 min), however, gustatory stimulation with lemon juice is more short-lived [24], and higher number of tablets of ascorbic acid or two instantly bitten tablets of ascorbic acid are likely to stimulate simultaneously more receptors than a single slowly dissolving tablet of ascorbic acid does [13]. Those differences between the ascorbic acid and the lemon juice and the amount of stimulant may have been responsible for these contradictory results.

The rest ADCs was higher in submandibular glands than in parotid glands and sublingual glands for both before and after RT. This finding was in accordance with previous studies [10, 14, 24, 40]. In the rest state, about two thirds of all saliva is produced by the submandibular glands, while the parotid glands produce less than one third [39]. Thus, the expected higher proportional amount of water in the extracellular space of the submandibular glands can explain the higher ADCs at rest [14]. The rest SFR in parotid glands was lower than the submandibular mixed with sublingual glands before RT also support this view. While the difference in rest ADCs may also be explained by the different histologic composition of the glands: the submandibular glands were mixed serous with mucous gland, whereas the parotid glands were purely serous [39]. Furthermore, the other possible contributing factor to the lower ADCs of the parotid glands is that adipose tissue in the parotid glands is higher than the submandibular glands [39]. The DWI technique relies on a fat-suppression technique, which can result in an overall decreased signal intensity of fatty tissues for all b factors.

In our study, the stimulated ADCs IRs in parotid glands was higher than in submandibular glands before and after RT respectively, which in accordance with the fact that most of the saliva is produced by the parotid glands in the stimulated state. The result that the Tmax of parotid glands was earlier than the submandibular glands and the sublingual glands also supported it.

The rest and stimulated ADCs increased after RT as a result of radiotherapy damage. Most studies reported the same result that a rise in ADCs in patients with xerostomia [13–15, 36], which are accordance with our observations. Though the time of the follow-up MRI examinations is variable among all these studies (from a few weeks to several months after RT), it was concordantly suggested that the ADCs increased after RT. Acinar cells show early and late microscopic alterations in glandular tissue response to radiation, there are particularly changes indicative of cell death in the early phase, the hypo-vascularization, formation of fibrous tissue in the late phase [2, 41–43], which leads to an decrease in the cell density and a greater increase of free water in the extracellular space, the motion of free water more faster and the ADCs increased. The rest and stimulated SFR of parotid glands, submandibular mixed with sublingual glands and full three glands after RT were lower than those before RT also indicated that the salivary secretary function decreased after RT. In the stimulated state, the amount of saliva was more than the rest state. However, the absolute value of SFR CRs in parotid glands and the full three glands at rest were higher than that of stimulated state, which also indicated that the radiation induced the acinar cells damage. The rest and stimulated ADCs CRs in the three glands separately had no significance also support this view.

The reason why the ADCs IRs in sublingual glands was higher than in parotid glands and submandibular glands before and after RT separately was that in the stimulated state, the saliva induced by the stimulant accumulated at the bottom of the mouth, which closest to the sublingual glands, and the volume of sublingual glands was the smallest among the three major glands, those reasons resulted a greater stimulus intensity of the unit area in the sublingual glands.

The median follow-up time was 16 months, and it was known that the salivary glands function may recover up 5 years after RT [44]. The SFR IRs after stimulation of parotid glands and the full three glands after RT were significantly higher than that before RT, and the Tmax of the sublingual glands after stimulation was earlier than that before RT, suggesting the retention and even hyperactivity of the salivary function after radiation, and the ability to repair and reserve to some extent.

When regarding the cutoff value of the rest ADCs before and after RT predicting the xerostomia, to our knowledge, there is no report in previous studies. In our study, we calculated the cutoff value of changes in ADCs and SFR, which provides a quantitative basis for predicting the xerostomia after RT. The 0.19 g/min of the rest SFR between xerostomia and non-xerostomia groups was very close to clinical standard in diagnosing xerostomia [45], which also identified the accuracy of the swab method in saliometry. The area under the curve, sensitivity and specificity were high in all of cutoff value, indicating the feasibility of establishing prediction model based on large samples.

Finally, we analyzed the correlation between the ADCs and the SFR, the ADCs and the changes in ADCs and the XQ scores respectively. The rest ADCs in submandibular glands was correlated with the rest SFR of submandibular mixed with sublingual glands and the full three glands before RT, the stimulated ADCs was correlated with the stimulated SFR in parotid glands before RT, both of them suggested the rest ADCs can reflect the secretary function of the salivary glands. And the increased ADCs after RT corresponding to the decreased SFR also indicated that the ADCs can reflect the secretary function of the salivary glands.

Both of the rest ADCs of parotid glands and XQ scores, the ADCs IRs of sublingual glands and the XQ scores, the rest ADCs CRs and the XQ scores had a positive correlation, which provided some evidences for the ADCs in predicting the severity of the xerostomia after RT. But the other changes in ADCs and the XQ scores had no significance, considering the reason that the xerostomia caused by radiation injury to salivary glands is a complex of subjective and objective factors, which is associated with the decreased saliva, the changes in saliva composition and viscosity and the tolerance of the patients. The XQ scores have certain subjectivity, and the increase in ADCs after RT is influenced by many factors, resulting in the deficiency of correlation.

The present study has several limitations. First, the study population was relatively small, which only reflect the data of some people in a certain region. Second, this study only analyzed the salivary glands of NPC patients more than 1 year and within 3 years after RT, while the salivary glands function and xerostomia are dynamic process after RT, and IMRT may have a long term effect on the salivary glands function in NPC patients. Thus, we propose that a large sample in multicenter in patients with different cancers in head-and-neck after RT with multiple follow-up periods and a long follow-up time be conducted to establish a predictive model, which help to establish an objective standard of xerostomia induced by RT based on DW-MRI.

## Conclusions

The ADCs increased after RT both in rest and stimulated state, the rest and stimulated ADCs with lemon juice before and after RT correlate with the SFR and the XQ scores, suggesting that DW-MRI with transient gustatory stimulation is potentially useful to evaluate hypofunction of salivary glands and also is a useful tool for detection of xerostomia induced by radiation noninvasively and objectively in clinical practice.

## Supplementary information

**Additional file 1:** The patient characteristics.

## Data Availability

The datasets used and/or analysed during the current study are available from the corresponding author on reasonable request.

## Abbreviations

ADCs: Apparent diffusion coefficients
CRs: Change rates
DW-MRI: Diffusion-weighted magnetic resonance imaging
IMRT: Intensity-modulated radiotherapy
IRs: Increase rates
NPC: Nasopharyngeal carcinoma
ROIs: Regions of interests
RT: Radiotherapy
SFR: Salivary flow rates
SI: Signal intensity
XQ: Xerostomia questionnaire
