# Supplementary material for: Salivary gland function in nasopharyngeal carcinoma before and late after intensity-modulated radiotherapy evaluated by dynamic diffusion-weighted MR imaging with gustatory stimulation
Source: BMC Oral Health. 2019 Dec 21;19:288. doi: 10.1186/s12903-019-0951-x (PMC6925496; doi:10.1186/s12903-019-0951-x)
Supplement: Supplementary file 1 — Additional file 1: The patient characteristics. [file 12903_2019_951_MOESM1_ESM.docx]

The patient characteristics.

| Patient | Pathology | T | N | Overall stage | RT | CCT | Dose of primary site(Gy) | Mean planned parotid dose(Gy) | Mean planned submandibular dose(Gy) | Mean planned sublingual dose(Gy) | XQ |
| --- | --- | --- | --- | --- | --- | --- | --- | --- | --- | --- | --- |
| 1 | low differentiation squamous cell carcinoma | 2 | 0 | Ⅱ | IMRT | TAXOL+DDP | 70.40 | 36.89 | 63.01 | 43.33 | 57 |
| 2 | undifferentiated non-keratinized carcinoma | 2 | 2 | Ⅲ | IMRT | TAXOL+DDP | 70.40 | 37.02 | 63.05 | 43.59 | 53 |
| 3 | low differentiation squamous cell carcinoma | 2 | 2 | Ⅳa | IMRT | TAXOL+DDP | 70.00 | 34.89 | 62.33 | 43.01 | 45 |
| 4 | undifferentiated non-keratinized carcinoma | 3 | 2 | Ⅲ | IMRT | TAXOL+DDP | 70.95 | 34.77 | 62.13 | 42.58 | 44 |
| 5 | low differentiation squamous cell carcinoma | 1 | 2 | Ⅲ | IMRT | TAXOL+DDP | 68.00 | 34.08 | 62.34 | 42.96 | 20 |
| 6 | low differentiation squamous cell carcinoma | 2 | 2 | Ⅲ | IMRT | TAXOL+DDP | 70.40 | 34.13 | 62.90 | 41.74 | 25 |
| 7 | low differentiation squamous cell carcinoma | 1 | 2 | Ⅲ | IMRT | TAXOL+DDP | 70.40 | 34.19 | 58.35 | 32.56 | 46 |
| 8 | undifferentiated non-keratinized carcinoma | 2 | 0 | Ⅱ | IMRT | TAXOL+DDP | 69.30 | 34.17 | 58.32 | 32.53 | 46 |
| 9 | low differentiation squamous cell carcinoma | 1 | 0 | Ⅰ | IMRT | DDP | 66.70 | 38.55 | 61.71 | 40.34 | 51 |
| 10 | undifferentiated non-keratinized carcinoma | 2 | 2 | Ⅲ | IMRT | TAXOL+DDP | 70.40 | 38.61 | 61.91 | 40.33 | 45 |
| 11 | low differentiation squamous cell carcinoma | 2 | 0 | Ⅱ | IMRT | DDP | 72.00 | 33.26 | 58.63 | 33.51 | 20 |
| 12 | low differentiation squamous cell carcinoma | 2 | 2 | Ⅲ | IMRT | TAXOL+DDP | 70.40 | 33.27 | 58.65 | 33.53 | 18 |
| 13 | low differentiation squamous cell carcinoma | 2 | 2 | Ⅲ | IMRT | TAXOL+DDP | 66.00 | 35.23 | 61.99 | 42.89 | 47 |
| 14 | undifferentiated non-keratinized carcinoma | 1 | 2 | Ⅲ | IMRT | TAXOL+DDP | 70.40 | 35.11 | 61.35 | 42.25 | 44 |
| 15 | low differentiation squamous cell carcinoma | 3 | 2 | Ⅲ | IMRT | TAXOL+DDP | 68.80 | 35.45 | 61.03 | 42.35 | 50 |
| 16 | low differentiation squamous cell carcinoma | 2 | 2 | Ⅲ | IMRT | TAXOL+DDP | 70.90 | 35.23 | 60.35 | 41.99 | 48 |
| 17 | undifferentiated non-keratinized carcinoma | 2 | 3 | Ⅳa | IMRT | TAXOL+DDP | 66.00 | 34.12 | 59.88 | 41.23 | 46 |
| 18 | low differentiation squamous cell carcinoma | 3 | 3 | Ⅳa | IMRT | TAXOL+DDP | 70.40 | 35.2 | 60.38 | 41.98 | 42 |
| 19 | low differentiation squamous cell carcinoma | 4 | 2 | Ⅳb | IMRT | TAXOL+DDP | 67.20 | 33.59 | 59.24 | 37.98 | 30 |
| 20 | non-keratinized carcinoma | 4 | 3 | Ⅳa | IMRT | TAXOL+DDP | 70.40 | 33.55 | 59.20 | 37.95 | 29 |
| 21 | low differentiation squamous cell carcinoma | 1 | 1 | Ⅱ | IMRT | TAXOL+DDP | 67.20 | 32.99 | 58.99 | 38.02 | 25 |
| 22 | low differentiation squamous cell carcinoma | 2 | 2 | Ⅲ | IMRT | TAXOL+DDP | 70.00 | 33.33 | 59.63 | 38.11 | 23 |
| 23 | undifferentiated non-keratinized carcinoma | 2 | 1 | Ⅱ | IMRT | TAXOL+DDP | 68.80 | 33.01 | 59.33 | 39.11 | 24 |
| 24 | non-keratinized carcinoma | 2 | 3 | Ⅳa | IMRT | TAXOL+DDP | 70.40 | 32.11 | 58.03 | 38.41 | 20 |
| 25 | undifferentiated non-keratinized carcinoma | 3 | 1 | Ⅲ | IMRT | TAXOL+DDP | 70.40 | 35.14 | 61.23 | 41.06 | 37 |
| 26 | undifferentiated non-keratinized carcinoma | 4 | 0 | Ⅳa | IMRT | TAXOL+DDP | 70.40 | 35.23 | 62.03 | 40.77 | 34 |
| 27 | low differentiation squamous cell carcinoma | 4 | 2 | Ⅳa | IMRT | TAXOL+DDP | 70.95 | 35.79 | 61.77 | 41.32 | 61 |
| 28 | undifferentiated non-keratinized carcinoma | 1 | 1 | Ⅲ | IMRT | DDP | 70.95 | 35.96 | 61.94 | 41.57 | 55 |
| 29 | undifferentiated non-keratinized carcinoma | 2 | 3 | Ⅳa | IMRT | TAXOL+DDP | 69.96 | 33.23 | 60.11 | 40.85 | 43 |
| 30 | low differentiation squamous cell carcinoma | 2 | 1 | Ⅱ | IMRT | TAXOL+DDP | 70.95 | 34.01 | 61.11 | 41.02 | 39 |

CCT = concurrent chemotherapy; TAXOL = paclitaxel; DDP = cisplatin; F = female; M = male; T = tumor category; N = nodal category; XQ = xerostomia questionnaire scores
